# Successful treatment of Grover’s disease with upadacitinib: A case report

**DOI:** 10.1177/2050313X261463981

**Published:** 2026-08-16

**Authors:** Eshan Arora, Rylee Oosterhuis, Ashley E. Sutherland

**Affiliations:** 1 Dalhousie Medical School, Halifax, NS, Canada; 2 Division of Dermatology, Dalhousie University, Halifax, NS, Canada

**Keywords:** Grover’s disease, upadacitinib, pruritus

## Abstract

Transient acantholytic dermatosis, also known as Grover’s disease, is an acquired pruritic eruption commonly refractory to conventional therapies. We report a case of a 76-year-old male with Grover’s disease presenting with persistent pruritus and skin lesions despite multiple treatments. Upadacitinib 15 mg once daily was initiated off-label and the patient reported marked improvement in pruritus within 2 days of treatment and substantial reduction in lesion burden after 1 month. The etiology of Grover’s disease is unclear but is believed to be due in part to occlusion and damage to the sweat glands. Emerging responses to immunomodulatory therapies suggest immune dysregulation may contribute to select cases of Grover’s disease. Upadacitinib, a selective Janus kinase 1 inhibitor, reduces pruritus and inflammation-associated cytokine signaling and represents a plausible therapeutic strategy. To our knowledge, this is the first reported case of Grover’s disease successfully treated with Janus kinase inhibition.

## Introduction

Transient acantholytic dermatosis, also known as Grover’s disease, is an acquired pruritic eruption with variable reported incidence, characterized by papulovesicular lesions most commonly affecting the trunk of older white males.^1^ Although traditionally described as self-limited, many patients experience chronic or relapsing disease with substantial pruritus and impaired quality of life.^1,2^ The pathophysiology of Grover’s disease is poorly understood.^2^ Histologically, Grover’s disease demonstrates focal acantholysis and dyskeratosis, and commonly proposed triggers include heat, sweating, and xerosis, suggesting contributions from both epidermal barrier disruption and immune dysregulation.^1,2^

Management is tailored to symptom severity and persistence. First-line therapy typically includes topical corticosteroids and emollients to reduce symptom burden.^2^ For refractory disease, systemic corticosteroids, oral retinoids, and narrowband ultraviolet B phototherapy have been employed, targeting keratinocyte differentiation and broader immunomodulatory pathways.^1,2^ However, therapeutic responses are variable, high-quality evidence is limited, and many patients with Grover’s disease fail to achieve sustained remission with conventional therapies.^1,2^

Upadacitinib is an oral, selective Janus kinase 1 (JAK1) inhibitor that modulates downstream signaling within the JAK–STAT pathway, leading to reduced transcription of inflammatory mediators.^3^ It is currently approved by Health Canada for several immune-mediated diseases, notably moderate-to-severe atopic dermatitis in adults and adolescents,^4^ where it has been shown to rapidly reduce pruritus and inflammation.^5^ Given its targeted immunomodulatory effects and observed benefits in pruritic inflammatory skin conditions, JAK1 inhibition represents a rationally plausible off-label therapeutic consideration for recalcitrant inflammatory dermatoses, including Grover’s disease. To our knowledge, this is the first case of Grover’s disease successfully managed with upadacitinib or JAK inhibition.

## Case presentation

A 76-year-old man was referred for evaluation of a pruritic eruption that began 4 months prior. The eruption started on his trunk and spread to his extremities while sparing his head and neck. He had no prior dermatologic history. Previous ineffective treatments from his family physician included prednisone, super-potent topical corticosteroids, oral and topical antibiotics, oral and topical antifungals, antihistamines, and over-the-counter emollients.

Examination revealed widespread hyperkeratotic papules coalescing into plaques on his trunk and proximal extremities. A shave biopsy was performed on the chest for diagnostic purposes. He was prescribed a compounded cream of menthol 0.5% with camphor 0.5% in betamethasone valerate 0.1% to be applied twice daily. The biopsy results demonstrated foci of acantholytic dyskeratosis with underlying lymphocytic infiltrate consistent with Grover’s disease.

At a 6-month follow-up the patient reported inadequate itch control and the eruption appeared slightly worse with larger papules coalescing into plaques with central crusting on the upper trunk and bilateral arms. He was started on isotretinoin 10 mg once daily and tacrolimus 0.1% ointment to be used twice daily. A repeat shave biopsy from the upper abdomen was completed to confirm diagnosis which again showed findings consistent with Grover’s disease.

Following 4 months of isotretinoin, the patient continued to experience severe pruritus, which negatively affected his sleep quality, though his skin examination remained stable. Due to poor tolerability of the medication’s side effects, the isotretinoin dose was reduced to 10 mg every other day and triamcinolone acetonide 0.1% ointment was prescribed for twice-daily use. Follow-up was performed 1 month later with no clinical improvement in skin disease or pruritus. Given the significant negative impact of the disease on his quality of life, we initiated a trial of upadacitinib 15 mg orally once daily, which was initiated off-label.

The patient reported complete resolution of itching within 2 days of starting upadacitinib, and after 1 month of treatment, there was noticeable improvement in the skin lesions. Unfortunately, the patient ran out of the medication at one point and had missed dosing for 3 weeks, during which time his pruritus recurred. He was subsequently restarted on upadacitinib 15 mg once daily. Within 1 week of restarting treatment, the patient reported that his itching resolved again, and he observed continued skin improvement after 3 weeks (Figure 1). Of note, upadacitinib was very well tolerated with no reported drug-related adverse events since starting medication.

**Figure 1.**
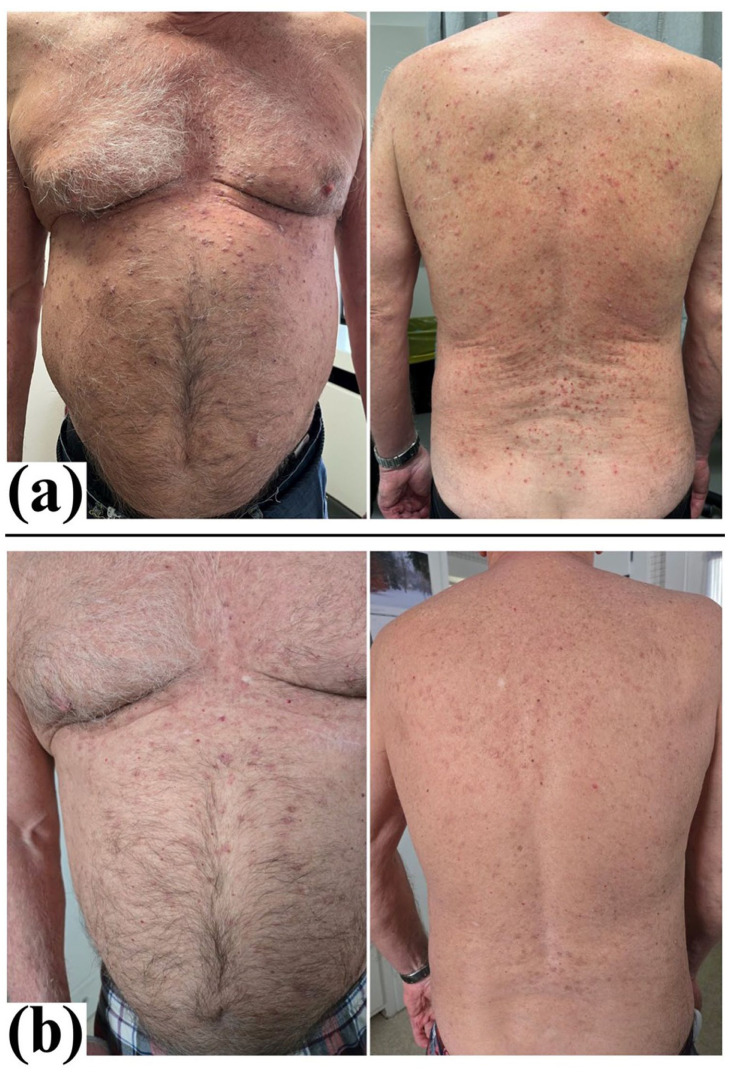
Erythematous papulovesicular lesions involving the anterior trunk and back before (a) and after 4 weeks (b) of therapy with upadacitinib 15 mg daily demonstrating marked reduction in lesion burden.

## Discussion

This case describes significant improvement of pruritus and skin lesions in a patient with Grover’s disease within days of treatment with upadacitinib. Grover’s disease can be challenging to manage when symptoms become chronic or recurrent. Although corticosteroids, oral retinoids, and phototherapy are commonly used, responses are inconsistent, and a proportion of patients continue to experience inadequate control.^1,2^ In recent years, a growing number of reports have described the successful off-label use of systemic immunomodulatory agents in refractory Grover’s disease, including dupilumab,^6,7^ naltrexone,^8,9^ and methotrexate,^10^ illustrating that alternative therapeutic strategies may be required for some patients.

Historically, Grover’s disease has been attributed to mechanical injury, sweat duct obstruction, and heat-related epidermal damage.^1,2^ However, accumulating clinical observations such as improvement with dupilumab and steroid-refractory disease courses suggest that immune-mediated pathways may contribute in a subset of patients.^6,7^ Together, these findings support the possibility that type-2 inflammatory signaling and pruritus-associated cytokines may play a role in chronic disease persistence.^11^ In particular, it has been hypothesized that Th2 cytokines may disrupt keratinocyte adhesion, potentially through up-regulation of tissue plasminogen activator, thereby contributing to acantholysis in susceptible patients.^12^

Upadacitinib selectively inhibits JAK1 and thereby modulates the JAK-STAT signaling pathway, reducing downstream transcription of pruritus and inflammation-related cytokines.^4,5^ Given the potential role of immune dysregulation in chronic Grover’s disease, JAK1 inhibition represents a biologically reasonable therapeutic approach in patients who exhibit persistent pruritus and inflammation despite conventional therapy.

Beyond its approved indications, upadacitinib is increasingly being used off-label for other immune-mediated dermatoses, including chronic eczematous dermatoses, bullous pemphigoid, hidradenitis suppurativa, and alopecia areata.^13^ In atopic dermatitis, upadacitinib has demonstrated earlier significant reductions in pruritus when compared with dupilumab, supporting the potential for more rapid therapeutic onset in highly pruritic conditions.^14^ The safety profile of upadacitinib has been well documented and needs to be considered in all patients prior to therapy initiation.^4,15^

In our patient, upadacitinib provided rapid (⩽3 days) and sustained improvement of symptoms, offering a steroid-sparing option with meaningful symptom relief although residual lesions remained. Future studies are needed to determine if this is an effective and safe treatment option in patients with Grover’s disease.

In summary, emerging reports of immunomodulator-responsive Grover’s disease suggest that targeted immune-based strategies may have a role in carefully selected patients. Our case adds to this evolving literature by suggesting that JAK1 inhibition may warrant further study as a potential therapeutic option in recalcitrant disease.
